# Mastectomized women’s perception of breast cancer early detection

**DOI:** 10.1371/journal.pone.0206405

**Published:** 2018-11-07

**Authors:** Indara Cavalcante Bezerra, Raimunda Magalhães da Silva, Cleoneide Paulo Oliveira, Christina César Praça Brasil, Mardênia Gomes Ferreira Vasconcelos, Marli Vilela Mamede, Marnewton Tadeu Pinheiro de oliveira

**Affiliations:** 1 Programa de Pós Graduação em Saúde Coletiva, Universidade de Fortaleza, Fortaleza, Ceará, Brazil; 2 Departamento de Fisioterapia, Centro Universitário Estácio de Sá, Fortaleza, Ceará, Brazil; 3 Programa de Pós Graduação em Saúde Coletiva, Universidade Estadual do Ceará, Fortaleza, Ceará, Brazil; 4 Departamento de Enfermagem, Universidade de São Paulo, Ribeirão Preto, São Paulo, Brazil; 5 Departamento de Tumores Cutâneos, Hospital São Camilo Cura D’Ars, Fortaleza, Ceará, Brazil; Jordan University of Science and Technology, JORDAN

## Abstract

**Background:**

A third of new cases of breast cancer could be detected early, which would prevent more serious consequences, such as mastectomy and death. Access to the subjectivity of mastectomized patients becomes relevant to elucidate failures in early detection of breast cancer and thus improve the cancer care network. Given that, the present study aimed to identify mastectomized women’s perception of the quality of care provided by the cancer care network for the early detection and diagnosis of breast cancer.

**Methods:**

Qualitative study carried out at a public outpatient cancer center in the city of Fortaleza, Ceará, Northeastern Brazil, to analyze the perceptions of 26 women who had undergone mastectomy after breast cancer based on Symbolic Interactionism.

**Results:**

The thematic analysis showed how women (re)structure their lives in the face of the structural and social aspects of coping with breast. Two essential themes emerged: “Contradictions regarding access to primary health care services and obstacles to the organization of SUS formal care network services” and “The informal and private health care network increase quality care coverage”.

**Conclusions:**

The absence of effective measures in Primary Health Care and patients’ ‘pilgrimage’ in the formal health care network have delayed early detection breast cancer.

## Introduction

Worldwide breast cancer (BC) rates are striking. Breast cancer accounts for a quarter of all cancers in women and nearly half a million deaths each year worldwide. In Brazil, 57,960 new cases were expected for the 2016/2017 biennium, with an estimated risk of 56.20 cases per 100,000 women [1–3].

The wide range of factors determining and conditioning breast cancer in women, in addition to the treatment, socioeconomic factors and characteristics of the health care system/team, have reoriented care practices within Brazil’s National Health System: the Unified Health System (*Sistema Único de Saúde–SUS*). Compliance with health guidelines is an important predictor of positive clinical outcomes, reducing mortality, hospitalizations and treatment costs. The recognition of individual determinants favors the development of intervention and support strategies that are fundamental to the management of breast cancer and to the improvement of the quality of care, which results in successful resolution of the patients’ condition, thus improving their quality of life [4].

Tackling breast cancer is challenged by the factors conditioning adherence to preventive and therapeutic procedures. At least one third of new cases of cancer could be detected early, which would avoid more serious consequences, such as mastectomy and death^5^.

However, an early diagnosis depends on the quality of health services, particularly Primary Health Care services. Quality care allows the diagnosis of the disease at an initial stage. The assessment of tumor location and its classification according to the extent, the degree of involvement and the presence of metastases determine cancer staging [5–8].

Breast cancer screening can result in better prognosis of the disease and reduced mortality, with less radical and more effective treatments, as well as lower rates of morbidity associated with therapy [9–10]. Mammography is the modality of examination used in screening for breast cancer [11–12].

Developed countries that implemented effective screening programs, with coverage of women within the target age range, quality examinations and adequate treatment, reduced breast cancer mortality rates by 20% to 30% by offering mammography screening [13]. This measure is cost-effective, but requires well-structured management tools and health systems. Although there has been an increase in breast cancer mortality rates worldwide, developed countries have reduced breast cancer mortality, mainly due to early detection of the disease and access to treatment. In Latin America and the Caribbean, mortality rates have been increasing slowly [13–16].

Deandrea et al. [17] conducted a study in the European Union (EU) and found that breast cancer screening programs based on routine mammograms were in place in 27 countries in 2014. In eight out of the 28 EU member states, the target age range was broader than that proposed by the EU, and in only three countries the full range was not covered. Fifteen countries reported not reaching some vulnerable populations, such as immigrants, prisoners and people without health insurance, while 22 reported that participation was periodically monitored by socioeconomic variables.

In Japan, 80% of the women within the target age range periodically undertake mammograms. The time and distance required to access cancer screening were viewed as factors that lower the participation rates in the 20% of the women who do not undertake mammograms [18].

Peppercorn et al. [19] have reported that 98% of American women are aware of the importance of mammography in the early detection of breast cancer. The authors point out that women living in rural areas of the United States receive mammography screening only when they have health insurance. Abraído-Lanza et al. [20] studied Latino women living in the United States and found that cultural and logistical barriers lower breast cancer screening participation rates and suggested intensifying public health campaigns targeted at this public.

Shirazi, Shirazi and Bloom [21], in a study of Afghan immigrant women living in the USA, have reported the need for a culturally competent faith-based health education framework to promote breast cancer screening among this population.

In Tajikistan, one of the poorest countries in Central Asia, almost half of the adult women have never heard of breast cancer. Access to diagnostic resources is often limited and screening mammography is neither feasible nor accessible to most of these women. The health system is predominantly hospital-based and focuses on curing patients, with low investments in health promotion and disease prevention. This context contributes to the evolution of the disease, making breast cancer one of the most common causes of cancer mortality in women in Tajikistan [22].

Importantly, two strategies are essential to reduce breast cancer mortality: early diagnosis and screening. Early diagnosis seeks to identify early-stage lesions by investigating signs, symptoms or risk factors for the disease and requires people’s and health professionals’ awareness. Screening aims to identify early-stage cancerous or noncancerous non-palpable lesions in people with no symptoms or clinical manifestations of the disease [12,23]. Public health policies and the allocation of resources targeted at the early detection of breast cancer determine the cure and the quality of life of these women.

In this regard, quality care stands out as a satisfactory response to users seeking health care needs. It is based on a broad perspective on care that seeks to alleviate suffering and promote health. It refers to the ability of health systems to solve patients’ problems at each level of care regardless of complexity and technological limitations. Quality care actions positively modify people’s perception of the health care reality and is an important determinant of the type of cancer evolution and prognosis [24–27].

Given the impact of breast cancer and the importance of the provision of quality care by health services, the following question should be asked: what do women who have undergone mastectomy think about the quality of care provided by the cancer care network?

The present study aimed to identify mastectomized women’s perception of the quality of care provided by the cancer care network for the early detection and diagnosis of breast cancer in a large city in Northeastern Brazil.

## Methodology

The present qualitative study was carried out on the basis of Symbolic Interactionism by applying a dialectical approach to aspects of social structuring and of the construction of individualities, apprehending the contradictions of contemporary life in the context of plural societies and seeking to access mastectomized women’s subjective accounts of the quality of the health care network [28].

According to symbolic interactionists, researchers need to understand the meanings given by the participants to experiences lived in a particular context in order to achieve a full understanding of the social process. This idea is based on three main premises. The first is that the humans act towards things on the basis of the meanings they ascribe to those things. The second is that the meaning of those things is derived from the social interaction that one has with one’s fellow. The third is that these meanings are handles in and modified through an interpretative process used by the person in dealing with the things they encounter [28].

The present study was carried out in an outpatient cancer care center in the city of Fortaleza, Ceará, Northeastern Brazil. The center performs circa 1400 consultations every month and at least 200 surgeries on women, 60% of which are mastectomies [29]. The center is one of the 27 cancer care facilities in the city–seven of these facilities (five hospitals and two outpatient centers are part of SUS).

Participants were 26 women who had undergone unilateral or bilateral mastectomy due to breast cancer at least 6 months prior to the study who were attending the center. Anonymity was protected by identifying the participants’ accounts using the letter ‘W’ (woman) followed by the numbers 1 through 26 according to the order in which they were interviewed.

Semi-structured interviews were carried out with the participants from December 2015 to January 2016. The questions addressed the meaning of the detection and diagnosis of breast cancer and the quality of cancer care provided by the national health care system. Filed notes and participant observation complemented the collected data.

Thematic analysis [30] was carried out on the basis of symbolic interactionism, which allows a dialectical analysis of how women (re)structure their lives in the face of the structural and social aspects of coping with breast cancer. These aspects are related to the meanings derived from the social interaction and from the interpretation of the lived objective and subjective reality. This premise enabled women to interpret and express the meaning of breast cancer in their lives based on the redefinition of experiences. Such “meaning” is understood herein as a socially and culturally constructed product [31].

The analysis was carried out by the researchers in a collective discussion following the procedures recommended by Minayo (2010): data ordering (organization of the empirical material), classification (horizontal and exhaustive reading of interviews and transversal reading), and final analysis (horizontal synthesis, vertical synthesis, and information confrontation by grouping convergent, divergent and complementary ideas) [30]. This process was followed in order to identify the themes that emerged from the empirical material.

Women’s different interpretations of their experiences in the face of coping with breast cancer gave rise to two essential themes that emerged from the collected data as results of the thematic analysis: “Contradictions regarding access to primary health care services and obstacles to the organization of SUS formal care network services” and “The informal and private health care network increase quality care coverage”.

All the procedures performed in the present study involving human participants were in accordance with the ethical standards of the national research committee and with the 1964 Helsinki declaration and its later amendments or comparable ethical standards. Informed consent was obtained from all individual participants included in the study. This research was approved by the research ethics committee of the University of Fortaleza under Approval No. 618.818. All the participants were over 18 years old and received verbal and written information on ethical issues. All the participants signed a free informed consent form.

## Results and discussion

Most of the women who participated in the study were Catholics (92%) and aged 47–56 years old (73.08%). There was a predominance of White women (53.8%), low levels of education (2–5 years of study– 42.31%) and income below one minimum wage (53.8%). Half of the participants were married, and the majority were housewives (38.46%).

### Contradictions regarding access to primary health care services and obstacles to the organization of SUS formal healthcare network services

The meaning women give to the detection and diagnosis of breast cancer and the quality of health care in the SUS network are related to their lived experiences in the health services. Women’s accounts of these experiences were analyzed on the basis of Symbolic Interactionism, which allowed to assess their experiences with the received care.

Thus, when questioned about the quality of care provided by the SUS services, the participants described their journey in search of breast health care. According to the participants, the journey begins in Primary Health Care (PHC) services, specifically in Family Health Care (FHC) centers, which provide consultations, request exams, and carry out diagnosis of breast cancer and consequent referral to specialized services.

In Brazil, the SUS cancer network is responsible for early diagnosis and screening in PHC centers through the request of mammogram for women. This exam is performed in specialized care centers and after the results come out patients can return to the family physician in PHC centers. If the results show any radiological changes that may represent risk of breast cancer, the patient will be referred to specialized care services in secondary health care centers to perform a biopsy. If the diagnosis is confirmed, the woman is referred to tertiary health care centers for starting chemotherapy and/or surgical treatment [9].

In the midst of this journey, the singularities of each woman, the dynamics of health services and the health care system contradictions are brought out. Sometimes FHC centers provide fast quality care, but at other times the care may be hindered due to the delay and shortage of physicians.

I first sought a primary health care center […] because the service was always good. I never had any difficulties. I could go there any day or every day, […] they scheduled everything, and everything was alright. I did everything right. Then the doctor requested some exams and I took them. When the result came out I returned to the center. I was really scared after the doctor told me he had seen a lump and that it was cancer. Oh my God! I was knocked off my feet

(W3).

I first went to the primary health care center, but sometimes it takes a long time to get an appointment through SUS. You come once, twice. The first day I went there I could not see the doctor. I went there, but the doctor was not there. Then the appointment was scheduled again. I had to come back the other day for a consultation. I just got a consultation the third time I went there. It is quite difficult, a whole drama. Too much suffering. If all the professionals were available maybe the provision of care would be faster and easier and we would suffer less with the treatment

(W19).

Quality care depends on the intersection of the patient journey and the way healthcare teams and services are organized–practices should be based on cohesion, that is, services should foster suitable conditions and training for the provision of care in addition to promoting patients’ cultural adaptation and the sharing of language and knowledge [25].

The context analyzed presents contradictions regarding access to breast cancer screening in PHC services, which compromises the quality of health care provided by the SUS network. However, the participants’ accounts point out that the diagnosis and treatment reflect on women’s sociopsychological aspects.

Azevedo and Costa [32] affirm that PHC should address the non-urgent health needs of the general population. The request for exams is complementary to this level of care. It is through the referrals that people can have access to services featuring better technologies, such as laboratories and specialized consultations.

With regard to the search for care in the SUS network, the participants emphasized obstacles to performing exams that are essential to the diagnosis of breast cancer. Aspects related to the municipal management and service delivery are thought to hinder access to specialized services and increase wait time for diagnosis.

[…] I was sent to the lab to perform examinations […] but they said that there was a problem, that the lab had not received any funding from the City Council and that there was only one analyst to disclose results. So, I had the biopsy in January, but in February they were still disclosing the results to the patients who had had the biopsy before me, back in December

(W25).

After the initial consultation in PHC, women find it difficult to access specialized services for diagnosis and treatment. Oliveira et al. [33] report that there is a poor distribution and shortage of specialized centers and professionals for diagnosis. There is a lack of a national program to carry out a regional and hierarchical monitoring of actions and sensitize health professionals to the early detection of breast cancer.

It should be noted, however, that access does not always ensure that the procedures will be carried out fast in the context of the services provided by PHC teams. Paim [34] criticizes this model and defines it as a “targeting policy” that does not differ from vertical programs, yet its conceptual model features innovative potentialities, such as comprehensiveness, effectiveness, technical quality, people’s satisfaction and the humanization of health services. These are major challenges for public policies in the field.

Inoperative and overwhelmed specialized health care network causes long queues. Many times when the patients receive the needed services the disease has already reached an advanced stage, with mastectomy being the only resource left to save their lives [35].

Another aspect highlighted in the patients’ accounts was the relationship between the health professional and the patient, especially the doctor’s conduct in communicating breast cancer diagnosis. According to some women, this relationship could be closer and built on the basis of good listening and care. Two participants emphasized professionals’ poor sensitivity to their complaints and symptoms before the diagnostic procedure.

I think the other doctor, the one in the primary health care center, should have found out about it some time ago. He was irresponsible, because he sees people as if they are nothing to him […] he just looked at the exams. He did not even look at my breasts. It was his duty to examine them. Then when I realized he was not going to examine them, I decided to tell him what I had. The professionals do not pay much attention to us or to what we are saying or feeling

(W2).

The doctor said it was little, but I used to touch it and feel it. It was not what he thought it was. I had this huge lump! And I said: Doctor, for God’s sake, give me a second chance! Check this! Let’s do another exam, another ultrasound, because the lump is here; I feel it, I can touch it with my hand. After much insistence he checked my breasts and requested an ultrasound. And that was it […]

(W15).

Oshiro et al. [36] have reported women’s accounts of health professionals’ disbelief in their complaints and symptoms reported during consultations. According to the women analyzed in their study, the physical examination, among other actions, can determine whether a doctor is interested or not, that is, a more detailed physical examination demonstrates the health professional’s interest in serving the patient. For women, this means greater accuracy in the detection and confirmation of the disease.

Women give great importance to prescriptions written and referrals made by the doctor. According to Turrini, Lebrão & Cesar [27], this behavior, which reaffirms the adoption of a biomedical model of care based on a ‘doctor-centered approach’, runs counter to proposals for reorienting health services and the changes brought about by the PHC proposal within the multidisciplinary care model.

A major assumption of symbolic interactionism is that meaning arises out of the process of interaction between people, resulting in possible ways of acting. That is, the search for possibilities for an interactive care in the field of breast health should take place in the first opportunity, and the health service should evaluate the potentialities of this encounter and the perceptions/impressions caused.

Akerman and Furtado [37] emphasize the high quality of care provided by reference centers for breast health care that prioritize the humanization of care for the detection, treatment, rehabilitation and reduction of breast cancer mortality.

The provision of quality care requires an integral approach to both individual and collective dimensions of health problems. It does not concern medical conduct or multidisciplinary actions, but it is closely related to access to appropriate diagnostic and treatment technologies [25].

Quality care presupposes breaking down dichotomies between primary health care actions and specialized care actions and is related to professional performance at all levels of care within the health care network.

### The informal and private health care networks increase quality care coverage

Social networks are defined as the voluntary web of relationships between people who exchange material, informational or emotional support. The most commonly examined aspect in friendship networks involving people with breast cancer has been the size of the network in terms of number of participating members as well as their potential to solve problems arising from the disease. Researchers have found that networks with greater social integration are closely associated with better survival and better quality of life after breast cancer [38].

In the field of breast health, the paths taken in search of care do not necessarily correspond to the flows determined by the formal health system. In order to access services, women often use personal influence and contacts, not following the PHC flow. The women analyzed in the present study confirmed this behavior:

I was examined right away because my daughter worked with a doctor. He was the one who put me here. I went to him first and then he sent me here to the ICC (specialized cancer care service). cancer care center because he said he worked here. And, by the way, he has been my doctor since then. I was very well taken care of. He expedited my surgery. Everything was very fast

(W19).

In addition to relatives and doctor friends, women have also been assisted by friendship networks in the search for early detection, diagnosis, testing and treatment:

The first exam I did was in Maracanaú (a nearby town). A friend of mine who works there helped me get it. It was faster because of that, because I knew someone who worked there, who helped me and put me ahead of the other patients. This person opened the doors for me

(W14).

A doctor who works at the ICC (specialized cancer care service) helped me to get a consultation. He used to go this Catholic church there every Thursday. He served people for free there and referred these people to other places. That’s how I got to the center. I was in pain and very weak; I was very skinny. Then he sent a letter to a friend of his who is a doctor at the ICC and everything worked out fine

(W2).

Women’s access to cancer detection, diagnosis and treatment in public health services is addressed in Brazil’s government documents, in the Federal Constitution of 1988, in the National Cancer Policy and in the National Breast Cancer Control Policy [39]. In these documents, the Government recognizes the importance of providing financial investment for the expansion of cancer care networks in order to provide comprehensive care to women with breast cancer based on SUS principles: universality, equity and comprehensiveness [33].

Nevertheless, the waiting list for specialized procedures, such as examinations and treatments, in health care facilities is considered critical. Rego and Nery [40] point out the lack of access to health care by people who do not have financial resources. However, access to breast health care by the women interviewed in the present study was facilitated by their friendship networks.

According to Symbolic Interactionism, group experience leads to self-directed and observable behaviors, i.e., in interaction people tend to engage in mutual help in such a way that they get to plan and direct their actions to help each other. The ‘other’ builds up the meaning of the objects of help used to make their own action plans [41].

Considering that the delay in diagnosis and treatment may be related to the numerous barriers encountered during the search for health care, it is possible to understand how the interactionist care provided by friendship networks can help overcome these barriers of access and avoid delays in breast cancer diagnosis and treatment [42].

It was harder for me to do the exams. I could only get to do the specific breast exams in fast way because a neighbor of mine who works here helped me. She expedited the whole process

(W4).

Women’s journey in search of breast health care within the public health system is often facilitated by their friendship networks.

Research carried out by Norsa’adah [43] emphasize that, in countries such as Malaysia, cancer patients pay for consultations, examinations and therapies to avoid delays in care when they face difficulties accessing breast cancer care. In Brazil, it is not different. As previously stated, women are aware of the delay in care in PHC and of the wait time for appointments with specialists or specific breast examinations.

The functioning of the public health system is inefficient and there is no guarantee of continuity of care. Therefore, being friends with health professionals or even paying for some examination or consultation determine the access to health care and the agility in patient referral, which does not comply with the formal patient flow and interferes with counter-referral [44].

A study of women from Sergipe (a city in Northeastern Brazil) demonstrated that they paid for imaging tests for breast lump detection and diagnostic confirmation [45]. They chose to pay for consultations and imaging tests as they realized it would be difficult to have access to tests through SUS and because they were aware of the delay in testing and results.

Thus, the quality of referral and counter-referral is called into question. Women get the necessary referrals, but they do not often reach the final service through SUS, thus having to pay for consultations and examinations.

I had to pay for mammography and ultrasound examinations myself. I preferred to pay for the blood test and all the other examinations so that I could get the results faster and have everything done right. I paid it all

(W23).

It is unsettling to think that most of these women belong to the less favored and underprivileged social classes, which suggests the need for well-functioning public health services, i.e., these women should have universal, comprehensive and equitable access to all the levels of care that guarantee the effective detection, diagnosis and treatment of cancer at an early stage [46].

The comprehensiveness and quality of SUS health care services should be reviewed so as to improve the provision of care to women with breast cancer in a timely manner. Legal guidelines have contributed to the analysis and production of innovations within Brazil’s national health system. However, the public-private interface has been routinely used by women in the search for breast cancer diagnosis and treatment [33].

Women’s accounts of their search for care highlight the indignity of the painful pilgrimage and the lack of options resulting from the misuse of public funds for other purposes. Governors remain indifferent to the suffering of women and to the fact that time determines the disease prognosis. That said, health care has been turned into a commodity [47].

## Conclusions

The absence of effective measures in the PHC network to sensitize women to preventive breast care and patients’ pilgrimage in the formal health care network have delayed early detection of breast cancer and led women to breast amputation, thus resulting in an irreparable biopsychosocial injury.

SUS has failed to provide effective breast cancer screening. Therefore, there is a need to involve the community, especially women, and direct therapeutic plans that focus on their singularities and respect the experience accumulated throughout life. Difficulties in access to specialized care, long diagnostic waiting time, failure to effectively integrate the services in the cancer network and professionals’ lack of communication and humanization training are the main barriers to care for women with breast cancer in SUS.

Faced with these challenges, many patients and their families–even those without any financial resources–feel pressured to pay for tests to accelerate treatment and resolve their health problem.

It should be noted that family and friends have been a positive option for getting fast diagnostic exams and access to specialized centers, which has not been reported in other studies. Importantly, these women should be provided with a broader solidarity network, which could improve the quality of care and the public-private interface. This network should comprise non-governmental and governmental organizations and social support groups in medium and high complexity services.

The findings of the present study point to the need for further research and actions, especially regarding information on breast cancer, strategies for early detection, and actions to minimize the delay in care and in the resolution of other problems within the cancer care network.
